# Extracellular Matrix Collagen Alters Cell Proliferation and Cell Cycle Progression of Human Uterine Leiomyoma Smooth Muscle Cells

**DOI:** 10.1371/journal.pone.0075844

**Published:** 2013-09-11

**Authors:** Faezeh Koohestani, Andrea G. Braundmeier, Arash Mahdian, Jane Seo, JiaJia Bi, Romana A. Nowak

**Affiliations:** 1 Department of Animal Sciences, University of Illinois, Urbana, Illinois, United States of America; 2 Wolfram Research, Champaign, Illinois, United States of America; University of Patras, Greece

## Abstract

Uterine leiomyomas (ULs) are benign tumors occurring in the majority of reproductive aged women. Despite the high prevalence of these tumors, little is known about their etiology. A hallmark of ULs is the excessive deposition of extracellular matrix (ECM), primarily collagens. Collagens are known to modulate cell behavior and function singularly or through interactions with integrins and growth factor-mediated mitogenic pathways. To better understand the pathogenesis of ULs and the role of ECM collagens in their growth, we investigated the interaction of leiomyoma smooth muscle cells (LSMCs) with two different forms of collagen, non-polymerized collagen (monomeric) and polymerized collagen (fibrillar), in the absence or presence of platelet-derived growth factor (PDGF), an abundant growth factor in ULs. Primary cultures of human LSMCS from symptomatic patients were grown on these two different collagen matrices and their morphology, cytoskeletal organization, cellular proliferation, and signaling pathways were evaluated. Our results showed that LSMCs had distinct morphologies on the different collagen matrices and their basal as well as PDGF-stimulated proliferation varied on these matrices. These differences in proliferation were accompanied by changes in cell cycle progression and p21, an inhibitory cell cycle protein. In addition we found alterations in the phosphorylation of focal adhesion kinase, cytoskeletal reorganization, and activation of the mitogen activated protein kinase (MAPK) signaling pathway. In conclusion, our results demonstrate a direct effect of ECM on the proliferation of LSMCs through interplay between the collagen matrix and the PDGF-stimulated MAPK pathway. In addition, these findings will pave the way for identifying novel therapeutic approaches for ULs that target ECM proteins and their signaling pathways in ULs.

## Introduction

Uterine leiomyomas (ULs) are one of the most common pelvic neoplasms in reproductive aged women with a reported prevalence of 25-70% depending on age [1–3]. These benign tumors originate from uterine smooth muscle cells (SMCs) and can cause severe symptoms such as abnormal uterine bleeding, pelvic pain and infertility [3]. Despite the prevalence of these tumors, there is limited understanding of their pathogenesis and few successful therapeutic strategies.

The most distinct feature of ULs is the excess synthesis and deposition of ECM proteins, mainly collagens type I and III [4–10]. Early studies by Stewart and Nowak [5] showed that collagen types I and III were both upregulated in ULs compared to normal myometrium. Recent global gene-profiling experiments have shown that ECM genes encoding collagen proteins are differentially expressed in ULs compared to normal myometrial SMCs [8,11–13]. In addition, ULs show alterations in the structure and composition of collagen fibrils, in that collagens are loosely packed and arranged in a nonparallel, disorganized manner [7]. There is also greater remodeling of the ECM in leiomyomas as they express higher levels of specific metalloproteinases (MMPs) including MMP2 and MMP11 [14–16]. These changes are contributing factors in the altered mechanical homeostasis in ULs leading to changes in cell signaling [17,18].

ECM collagens are known to both maintain cellular morphology and act as conduits between extracellular stimuli and cells by regulating proliferation, migration, differentiation, and survival [19]. The ultrastructure of fibril-forming collagens I and III has distinct effects on cellular morphology and proliferation mediated through focal adhesions and signaling pathways such as mitogen activated protein kinase (MAPK) [20–22]. Normal and malignant cells, such as fibroblasts, endothelial cells, hepatic stellate cells, vascular SMCs, bladder SMCs and melanoma cells all show an expanded morphology on monomeric collagen in contrast to a more dendritic morphology on fibrillar collagen [20–26]. A monomeric collagen matrix also stimulates cellular proliferation. Vascular SMCs and hepatic stellate cells cultured on polymerized collagen I fibrils show reduced cell proliferation in contrast to cells grown on monomeric, unpolymerized collagen [22,27]. These effects are likely modulated through growth factors such as PDGF since the ECM can act as a repository for growth factors changing their bioavailability and function [28–31].

Proposed mechanisms that may explain the growth modulatory effects of different forms of ECM collagens include interaction through integrins which are the main collagen receptors. Clustering and activation of integrin receptors induces cytoskeletal reorganization and formation of focal adhesions followed by activation of specific focal adhesion kinases (FAK). Activation of FAK then activates signaling pathways such as mitogen activated protein kinase (MAPK) and phosphatidylinositol 3-kinase (PI3K) pathways, altering the expression of cell cycle regulatory proteins and promoting proliferation [32–37]. Collagen matrices can also directly affect cell growth through interactions with discoidin domain receptors (DDRs) independent of cell spreading and cytoskeletal changes [37–39].

The fact that ULs are fibrotic tumors containing an abundance of disorganized ECM collagen [7,17] led us to investigate the pathogenesis of these tumors in the context of how these different forms of ECM collagen modulate LSMC behavior and how they interact with PDGF, a growth factor that is abundantly expressed in ULs. Using an *in vitro* model system of ECM collagen, we examined the interaction of cultured LSMCs with monomeric unpolymerized collagen films and fibrillar polymerized collagen gels in modulating cellular morphology, cell proliferation, cell cycle progression, and the associated signaling pathways.

## Materials and Methods

### Tissue collection and cell culture

Leiomyoma samples were obtained from premenopausal women undergoing hysterectomy at either Carle Foundation Hospital (Urbana, IL) or Feinberg School of Medicine, Northwestern University (Chicago, IL). Permission to use these samples was approved by the Institutional Review Board at the University of Illinois at Urbana-Champaign and Northwestern University. All samples were obtained after receiving written informed consent from the patients. Tissue samples were manually minced and then digested in Dulbecco Modified Eagle’s Medium (DMEM; Biowhittaker, Walkersville, MD) containing 1.5 mg/ml Collagenase for 4-6 hours at 37 degrees. Once digested, cells were cultured in DMEM medium containing 10% serum [DMEM supplemented with 5% FBS (Hyclone, Logan, UT), 5% BCS (Hyclone, Logan, UT), and 10,000 U pen/ml penicillin (Biowhittaker, Walkersville, MD), 10,000 µg strep/ml streptomycin(Biowhittaker, Walkersville, MD), and 200 mM L-glutamine(Biowhittaker, Walkersville, MD) from here on called 10% medium] at 37°C in a humidified atmosphere of 95% air and 5% CO_2._

### Collagen matrix preparation

PureCol collagen solution (Advanced Biomatrix, San Diego, CA) was diluted to 1.0 mg/ml with 0.1 M acetic acid at room temperature. For monomeric collagen coating, plastic dishes were incubated with 0.5 M acetic acid for 20 minutes at room temperature, rinsed once with distilled water and coated with diluted collagen under sterile conditions for 4-6 hours. For fibrillar collagen coating, diluted collagen was neutralized with 10x PBS, 0.1 N NaOH and 0.1 N HCl on ice. Plastic dishes were coated with fibrillar collagen and incubated at 37 degrees overnight for the gel to polymerize. Coated dishes were rinsed with PBS several times before seeding cells.

### Cell proliferation assays and flow cytometric analysis

To obtain the growth curve of LSMCs, equal numbers (100x10^5^) of trypsinized LSMCs from confluent dishes were cultured in 10% serum-containing medium on different matrices. Cells were trypsinized and counted every other day using a hemocytometer. We also used thymidine incorporation assays to measure the proliferation of LSMCs on different matrices in the absence or presence of PFGF-AB (R&D Systems, Minneapolis, MN). Briefly ,LSMCs were cultured at equal numbers (2000 cells) in 96-well plates coated with monomeric or fibrillar collagen. Cells from 3 patients with 6 experimental replicates per plate were used. For cell cycle synchronization, LSMCs at 90% confluence were serum starved for 24 hours and then treated with 10% serum medium, medium with no serum, or medium containing 10 ng/ml PDGF for 24 hours. Cells were labeled with 0.01 µCi/µl tritiated thymidine (PerkinElmer, Waltham, MA) for 18 hours after treatment. Cells were then trypsinized and harvested on filter papers for reading in a Wallace Microbeta liquid scintillation counter (Oy, Finland).

For flow cytometric analysis, leiomyoma cells were grown to confluency in 10% serum-containing medium. Once confluent, cells were washed with PBS and then placed in serum-free medium and serum starved for 24hrs. Following serum starvation, cells were washed and plated again in serum free medium or in serum free medium containing 10 ng/ml PDGF for 24hrs. At this time cells were harvested with 0.25% trypsin/EDTA, washed with PBS and fixed in 70% ethanol at -20°C overnight. Fixed cells were centrifuged and washed with PBS and stained with a staining solution containing 2 mM MgCl2, 1 mg/ml propidium iodine (Sigma, St. Louis, MO) and 1 mg/ml RNase A (Roche, Indianapolis, MN)/PBS overnight at 4°C. Stained cells were taken and analyzed for cell cycle phase distributions in a FACScan flow cytometer (Becton Dickinson, Franklin Lakes, NJ). All data were analyzed using FCS express v4 software (De Novo software, LA, CA).

### Cell adhesion assay

Adhesion of LSMCs to different matrices was compared in cells seeded onto either plastic, monomeric collagen or fibrillar collagen coated dishes. Three hours after seeding, dishes were gently rinsed with PBS to remove unattached cells and imaged to count the number of attached cells to each matrix. Adhesion assays were performed on cells from three different patients with two experimental replicates for each patient. The numbers of attached cells in 10 fields of 20 mm^2^ surface area were averaged and represented as mean ± SEM.

### Immunofluorescence microscopy

Immunofluorescence staining of cells was carried out by first fixing cells in 4% paraformaldehyde for 30 minutes at room temperature. Following three washes in PBS, cells were permeabilized in 0.1% triton X-100 for 15 minutes. Dishes were next incubated in ultra-cold methanol for 15 minutes at -20°C and washed for half an hour before blocking by Image-iT FX Signal FX Enhancer (Invitrogen, Carlsbad, CA) for 30 minutes. Following a PBS wash, cells were incubated with 1:100 dilutions of primary antibodies against vinculin (Sigma, St. Louis, MO), pFAK (Cell Signaling; Danvers, MA), and integrin α2 (Santa Cruz Biotechnology, Santa Cruz, CA) for 2 hours at room temperature. A 1:200 dilution of secondary antibodies conjugated with different fluorophore antibodies (Invitrogen, Carlsbad, CA) was applied to incubating cells for 2 hours. After three washes with PBS, cells were stained with 10 µg/ml DAPI (Invitrogen, Carlsbad, CA) for 15 minutes. Dishes were cured in Prolong Gold (Invitrogen, Carlsbad, CA) for 24 hours in darkness before imaging with a Zeiss LSM710 microscope (Oberkochen, Germany).

### lmmunoblotting

LSMCs were cultured on different matrices and treated with or without protein inhibitors (Sigma, St. Louis, MO) prior to treatment with 10 ng/ml PDGF. At the end of the treatment time, cell lysates were collected using RIPA lysis buffer containing protease cocktail inhibitors (Roche, Indianapolis, IN). BCA assays (Thermo Scientific; Rockford, IL) were performed on cell lysates to determine protein concentrations. Proteins were loaded onto 4-20% gradient SDS-PAGE gels (Thermo Scientific; Rockford, IL) under reducing conditions before being transferred to nitrocellulose membranes. Primary antibodies [anti-phospho-ERK1/2 (1:1000; Cell Signaling; Danvers, MA), anti-ERK1/2 (1:1000; Cell Signaling; Danvers, MA), phospho PDGF-Rβ (1:500; Cell Signaling; Danvers, MA), anti-PDGF-Rβ (1:500; Cell Signaling; Danvers, MA), phospho-AKT (1:1000; Cell Signaling; Danvers, MA). AKT (1:1000; Cell Signaling; Danvers, MA), RhoA (1:1000; Cell Signaling; Danvers, MA), GAPDH (1:2000; BD Transduction Laboratories, Lexington, KY), p21 Waf1/Cip1 (1:2000; Cell Signaling; Danvers, MA); p27 Kip1 (1:1000; Cell Signaling: Danvers, MA); cyclin E (1:1000; Cell Signaling: Danvers, MA); and cyclin D2 (1:1000; Cell Signaling: Danvers, MA).] were incubated at 4°C overnight. Incubation with HRP-conjugated secondary antibody (Cell Signaling; Danvers, MA) was for 60 min at room temperature (1:10,000) and HRP signal detection was carried out with Super Signal West Pico Chemiluminescent Substrate kit (Thermo Scientific; Rockford, IL). Membranes were first probed with anti- phosphorylated protein antibody and then stripped and re-probed with anti-total protein antibody as a loading control. ImageJ software from the National Institutes of Health was used for densitometric analysis.

### Statistical analysis

Regression analysis was performed to estimate the growth rate of LSMCs. Experimental variability between treatments/matrices was determined by an analysis of variance (ANOVA) model. To determine statistical differences between groups, *post-hoc* orthogonal comparisons were applied. Values are expressed as mean ± SEM and p< 0.05 was considered statistically significant. STATA SE (Version 11.2) software was used for conducting all statistical analyses.

## Results

### Morphology of LSMCs on different collagen matrices

**Figure 1:**
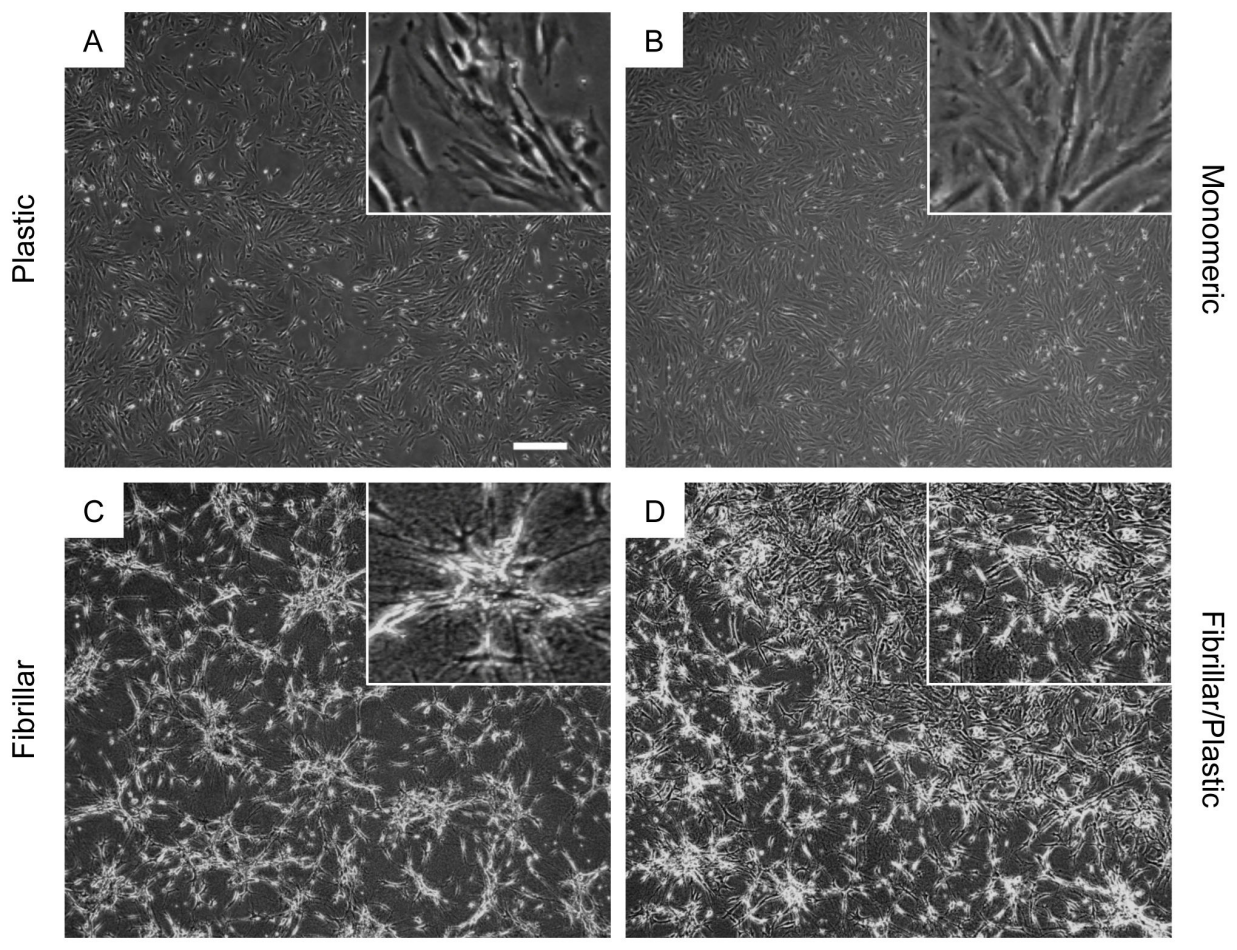
Morphology of LSMCs changes on different collagen matrices. **Equal numbers of** LSMCs were cultured on plastic (A), monomeric (B) or fibrillar (C) collagen-coated dishes in serum containing medium for 48 hours and then imaged. Three hours after seeding, the fibrillar collagen coating of a dish was partially removed to expose LSMCs to the plastic matrix (D) (Bars 200 µm in main figures; 50 µm in inserts).

To understand the interaction of LSMCs with different collagen matrices, we cultured primary LSMCs on plastic, monomeric collagen or fibrillar collagen-coated dishes and examined cell morphology 48 hours later. LSMCs on monomeric collagen-coated dishes displayed a spindle-like morphology typical of SMCs that was similar to LSMCs on control, plastic dishes (Figure 1A & B). In contrast, LSMCs on fibrillar collagen-coated dishes had a star-like morphology, with reduced cellular size and numerous cellular projections (Figure 1C). The star-like morphology was found to be reversible. When the fibrillar coating was removed from one section of the culture dishes by scraping with a sterile, plastic pipette tip the LSMCs returned to their spindle-like morphology and grew similarly to cells on plastic and monomeric collagen matrices (Figure 1D). Growth of LSMCs on fibrillar collagen was also slower than cells on plastic or monomeric collagen-coated dishes as observed by the differences in confluency of dishes 48 hours after initial seeding.

### Proliferation of LSMCs on different collagen matrices

**Figure 2:**
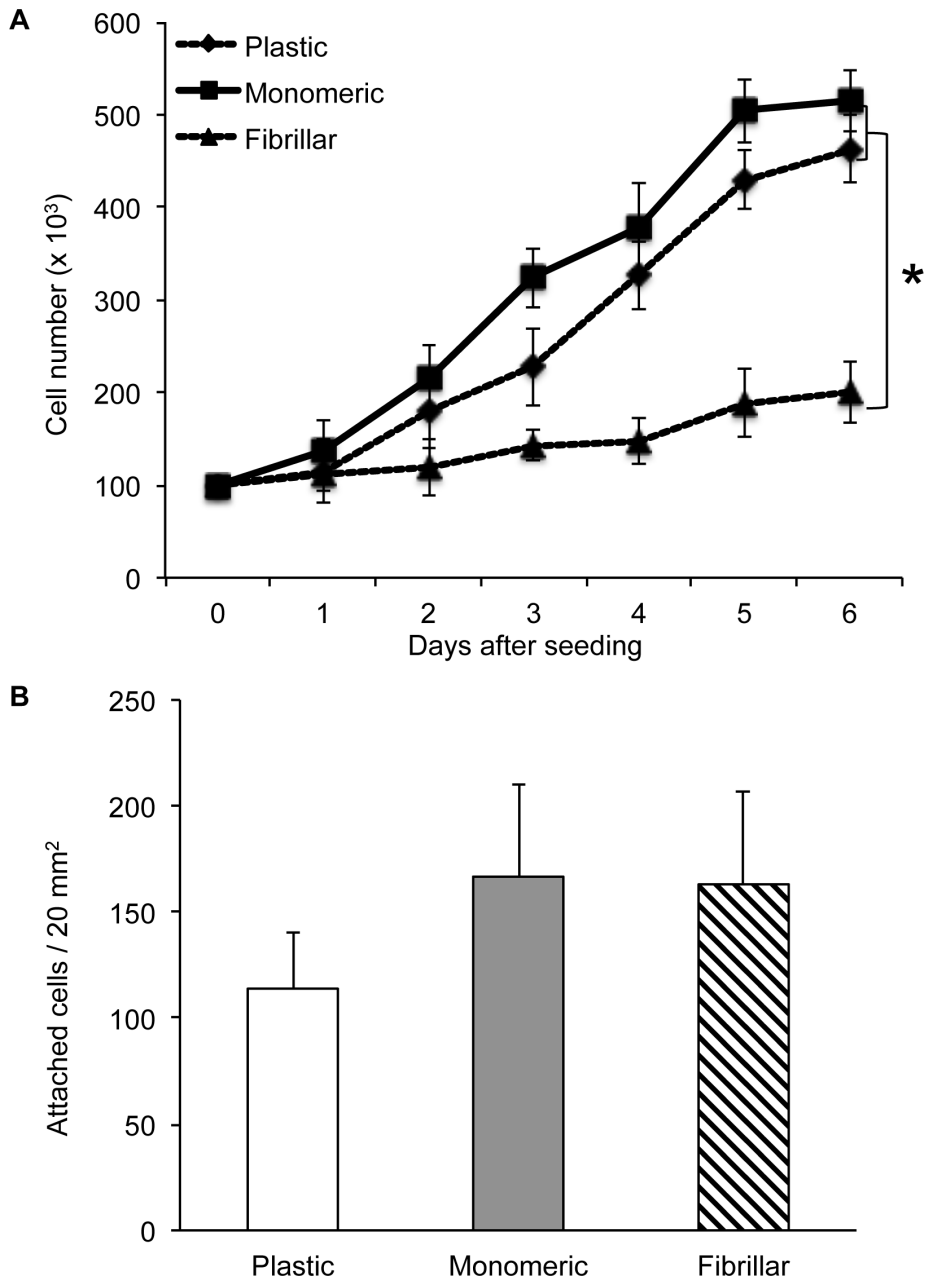
Growth, but not attachment of LSMCs is different on collagen matrices. (A) LSMCs were cultured on plastic, monomeric, or fibrillar collagen-coated dishes in serum-containing medium for 6 days. At the end of each day cells were trypsinized and counted using a hemacytometer. Rate of growth on each matrix was calculated using linear regression. (B) LSMCs cultured on different matrices were gently washed three hours after seeding and imaged to count the number of attached cells to each matrix. Statistical significance between matrices is indicated by asterisk (n=3, p<0.05).

To quantify LSMC proliferation on different collagen matrices, growth curves using individual cell counts and tritiated thymidine incorporation assays were performed. Growth curves for LSMCs cultured on collagen matrices in the presence of 10% serum showed that both monomeric and fibrillar collagen were permissive to cell proliferation. However, the rate of LSMC proliferation on monomeric collagen was significantly greater than for LSMCs cultured on fibrillar collagen (Figure 2A). The doubling time of LSMCs on monomeric collagen was 1.5 days compared to 6 days for cells cultured on fibrillar collagen. To confirm that differences in growth rates were not due to different adhesive properties of the cells on monomeric and fibrillar collagen, LSMCs were seeded on these matrices and allowed to attach for three hours before washing the unattached cells and counting the remaining attached cells. Results showed that there was no difference between the two collagen matrices in adhesion efficiency (Figure 2B) that could account for the difference in rate of cell proliferation. Therefore, the two forms of collagen differentially modulate the growth of LSMCs independent of their adhesion properties.

**Figure 3:**
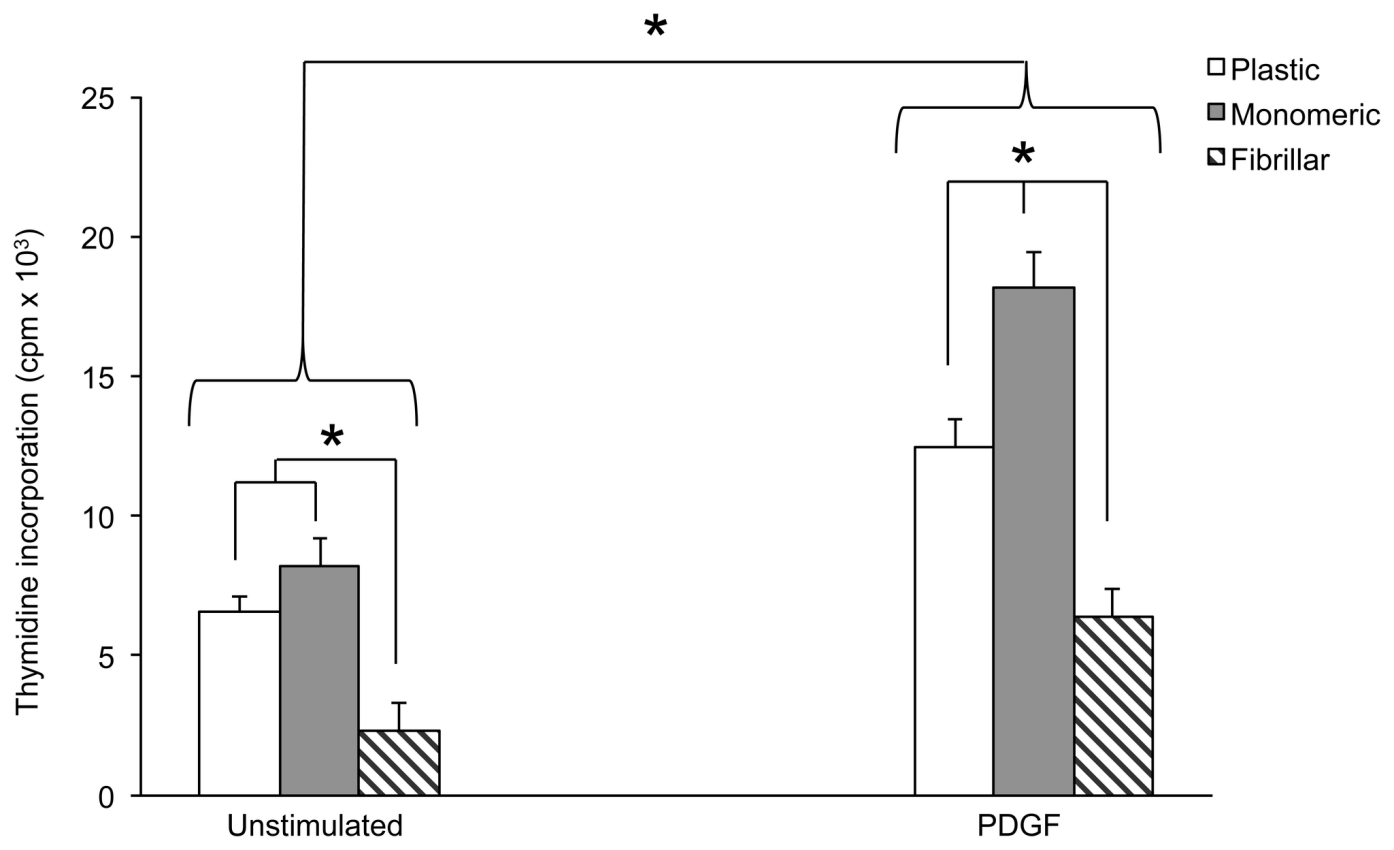
Basal and PDGF-stimulated proliferation of LSMCs are different on collagen matrices. Cell-cycle synchronized LSMCs were cultured on 96-well plates coated with different forms of collagen in medium with or without PDGF(10 ng/ml) for 24 hours. At the end of the treatment, cell proliferation was measured with thymidine incorporation assays. Statistical significance of the cellular growth rate between matrices within each treatment group as well as between stimulated and non-stimulated cells are indicated by asterisks (n=3, p<0.05).

LSMCs within leiomyoma tissues are exposed to many growth factors including PDGF which can exist bound to the ECM. We next examined whether the interaction of LSMCs with ECM collagen alters the way these cells respond to the stimulatory effects of PDGF. Basal and PDGF-stimulated proliferation of LSMCs cultured on either monomeric or fibrillar collagen matrices for 24 hours was measured. Results showed that, similar to our growth curve analysis, both matrices were permissive to proliferation of LSMCs in the presence or absence of PDGF with monomeric collagen having a significant potentiating effect over fibrillar collagen (Figure 3). Interestingly, PDGF treatment further increased LSMC proliferation on monomeric collagen compared to plastic suggesting a synergistic effect between the monomeric, unpolymerized collagen and PDGF. LSMCs grown on fibrillar collagen were responsive to the stimulatory effects of PDGF (40% increase) although to a lesser extent than cells grown on monomeric collagen (60% increase) when compared to non-stimulated cells.

**Figure 4:**
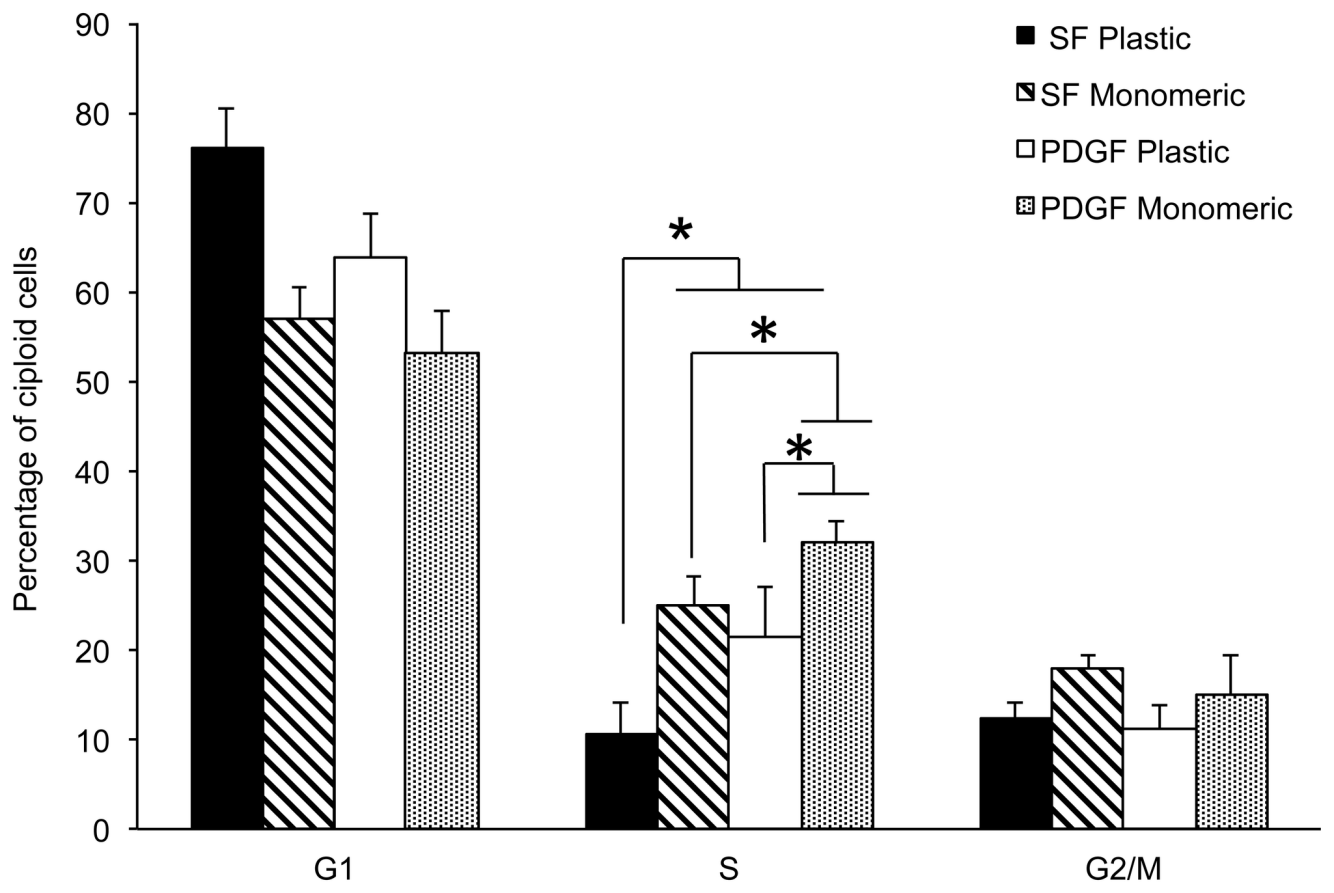
Effect of ECM collagen on cell cycle progression in LSMCs. Leiomyoma cells were treated with serum free (SF) medium or PDGF (10ng/ml) for 24 hours on either plastic or monomeric collagen matrices and then assayed by FACs analysis for cell cycle phase. 10,000 events were counted per cell line and the percent of cells in each phase is expressed as total number of diploid cells. Statistical analysis by orthogonal contrast statements was performed between treatments in the S phase of the cellular cycle to address changes in cellular proliferation. Asterisks denote statistical significance of the indicated comparisons (n=6; p<0.05).

**Figure 5:**
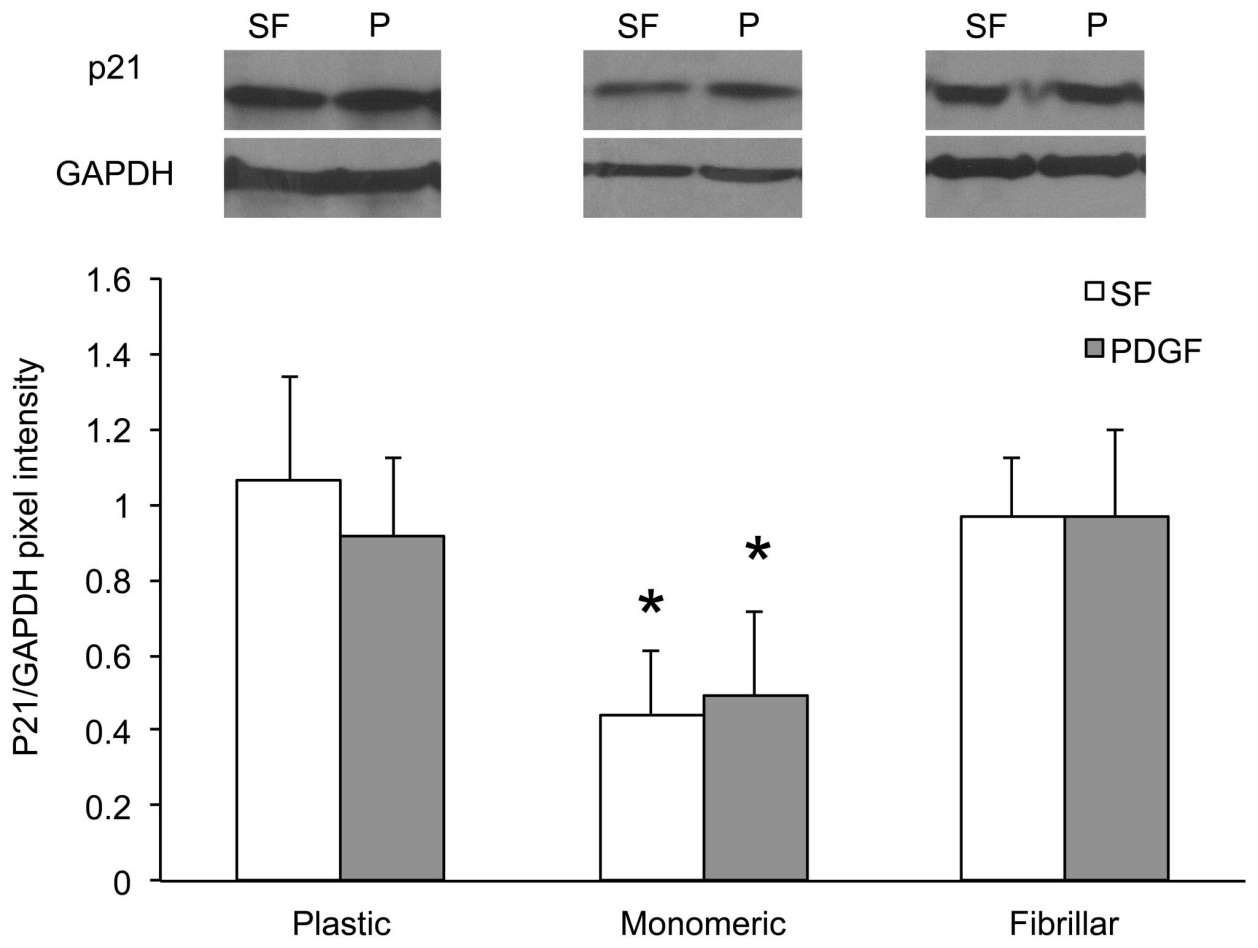
Effect of ECM collagen on p21 expression in LSMCs. Leiomyoma cells were treated with serum free (SF) or PDGF (10ng/ml) for 24 hours on either plastic, monomeric or fibrillar collagen matrices. Cell lysates were subjected to SDS-PAGE and immunoblotted for p21and GAPDH expression. Densitometry values were normalized to GAPDH expression and compared amongst all treatments and matrices. Statistical analysis by orthogonal contrast statements was performed comparing each treatment within each matrix and also comparing each treatment across all matrices. Statistical significance for all comparisons is indicated by asterisks (n=4; p<0.05).

To determine whether the stimulatory effects of monomeric collagen on the growth of LSMCs was the result of cell cycle modulation, cells were cultured on plastic or monomeric collagen-coated dishes for 24 hours in the presence or absence of PDGF and the distribution of DNA content was measured by flow cytometry (FACS). Due to the difficulties in separating LSMCs from the fibrillar collagen gels we were unable to obtain single cell suspensions required for FACs analysis for this treatment group. In support of the proliferation studies on plastic and monomeric collagen, FACs analysis showed that treatment of LSMCs with PDGF increased the percentage of cells in the S phase of the cell cycle compared to non-stimulated cells (Figure 4). Culture of LSMCs on monomeric collagen further increased the progression of cells through the cell cycle. To explain the change in cell cycle progression we analyzed p21 (an inhibitor of cell cycle progression) expression by immunoblotting of LSMCs cultured on plastic, monomeric or fibrillar collagen matrices. When normalized to GAPDH expression, p21 expression was lowest in LSMCs cultured on monomeric compared to plastic or fibrillar collagen matrices (Figure 5). Therefore, monomeric collagen may promote cell cycle progression by down-regulating endogenous levels of p21 thereby removing the “regulatory brake” for cellular proliferation. We also examined expression of p27, cyclin E and cyclin D2 but were unable to show a consistent effect of the different matrices on these cell cycle regulatory proteins (data not shown). We did not see a difference in p21 levels in LSMCs cultured on plastic or fibrillar collagen even after stimulation with PDGF (Figure 5.). There are two explanations for these findings. First, PDGF stimulation of cell proliferation (Figure 4.) may not involve regulation of p21 but rather stimulation of LSMCs to move through the cell cycle by possibly upregulating other CDK proteins and second, fibrillar collagen may not inhibit LSMC proliferation through p21 but through other cell cycle inhibitors.

### Localization of cytoskeletal F-actin and focal adhesion components in LSMCs cultured on different collagen matrices

**Figure 6:**
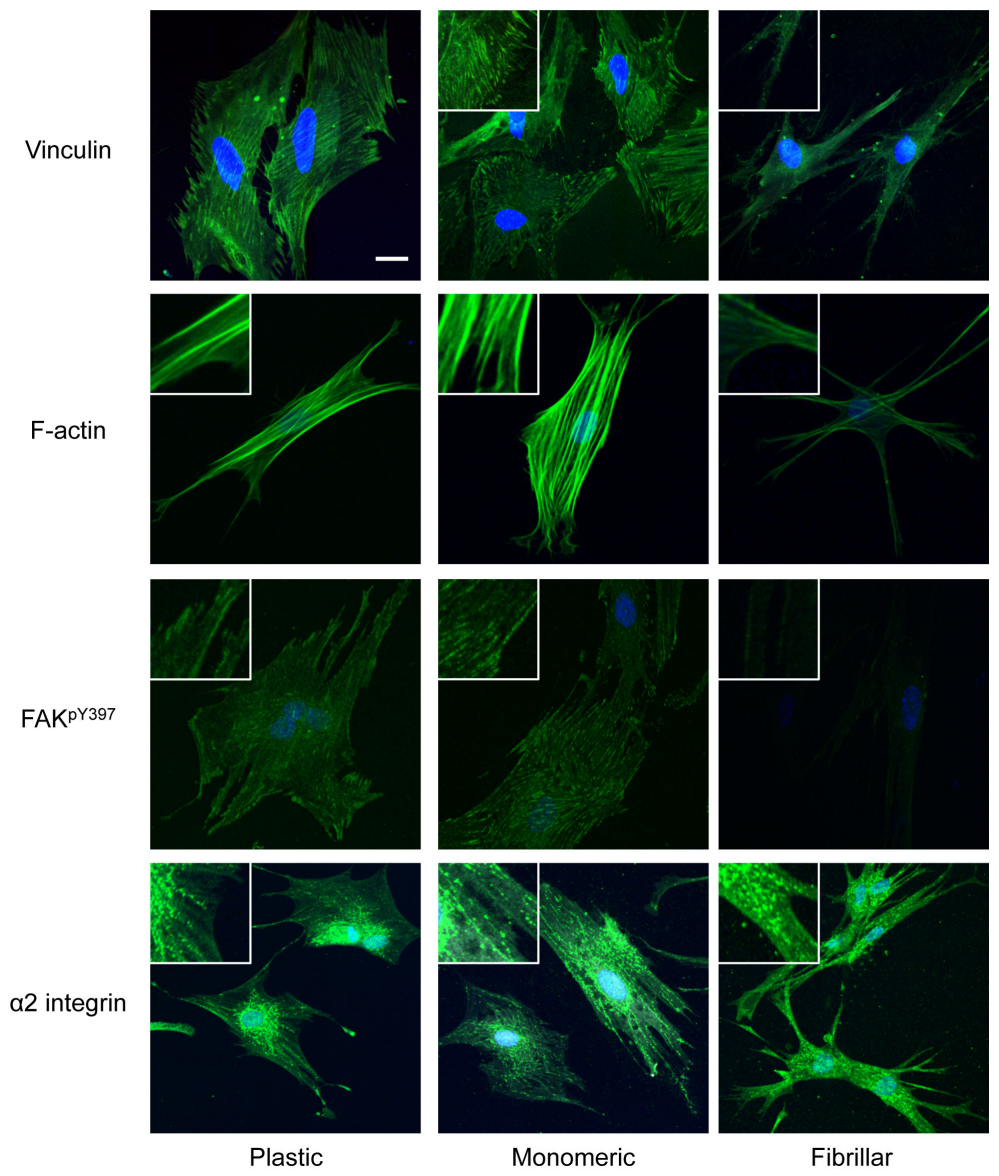
Activation of focal adhesion complexes and F-actin stress fibers in LSCMs is altered on different collagen matrices. LSMCs were cultured on different matrices for 24 hour and then fixed and stained with DAPI and specific antibodies against FAK pY397, α2 integrin, vinculin, and F-actin for imaging (Bar 50µm).

ECM exerts its effects on cells through the activation of several mediatory proteins including integrins, FAK and vinculin leading to the assembly of cytoskeletal components. To investigate whether the differential proliferative responses of LSMCs cultured on monomeric and fibrillar collagen matrices occur through such mediatory proteins, we immunostained LSMCs cultured on collagen matrices for α2β1 integrin, phospho-FAK (pY397), vinculin, and F-actin stress fibers. Results showed no difference in abundance or clustering of α2β1 integrin across matrices whereas LSMCs cultured on monomeric collagen displayed abundant activation (phosphorylation) of FAK, specifically at the sites of matrix adhesion (Figure 6). In contrast, LSMCs cultured on fibrillar collagen showed minimal FAK phosphorylation at Y397 throughout the cell cytoplasm. Localization of vinculin in LSMCs on monomeric collagen showed a punctate pattern which differed from the more widespread localization observed in cells grown on fibrillar collagen (Figure 6). Formation of F-actin stress fibers in LSMCs grown on monomeric collagen was quite pronounced with distinct lines of central and peripheral fibers compared to the presence of thin, lightly stained peripheral fibers in cells grown on fibrillar collagen (Figure 6).

### Activation of MAPK signaling pathway

**Figure 7:**
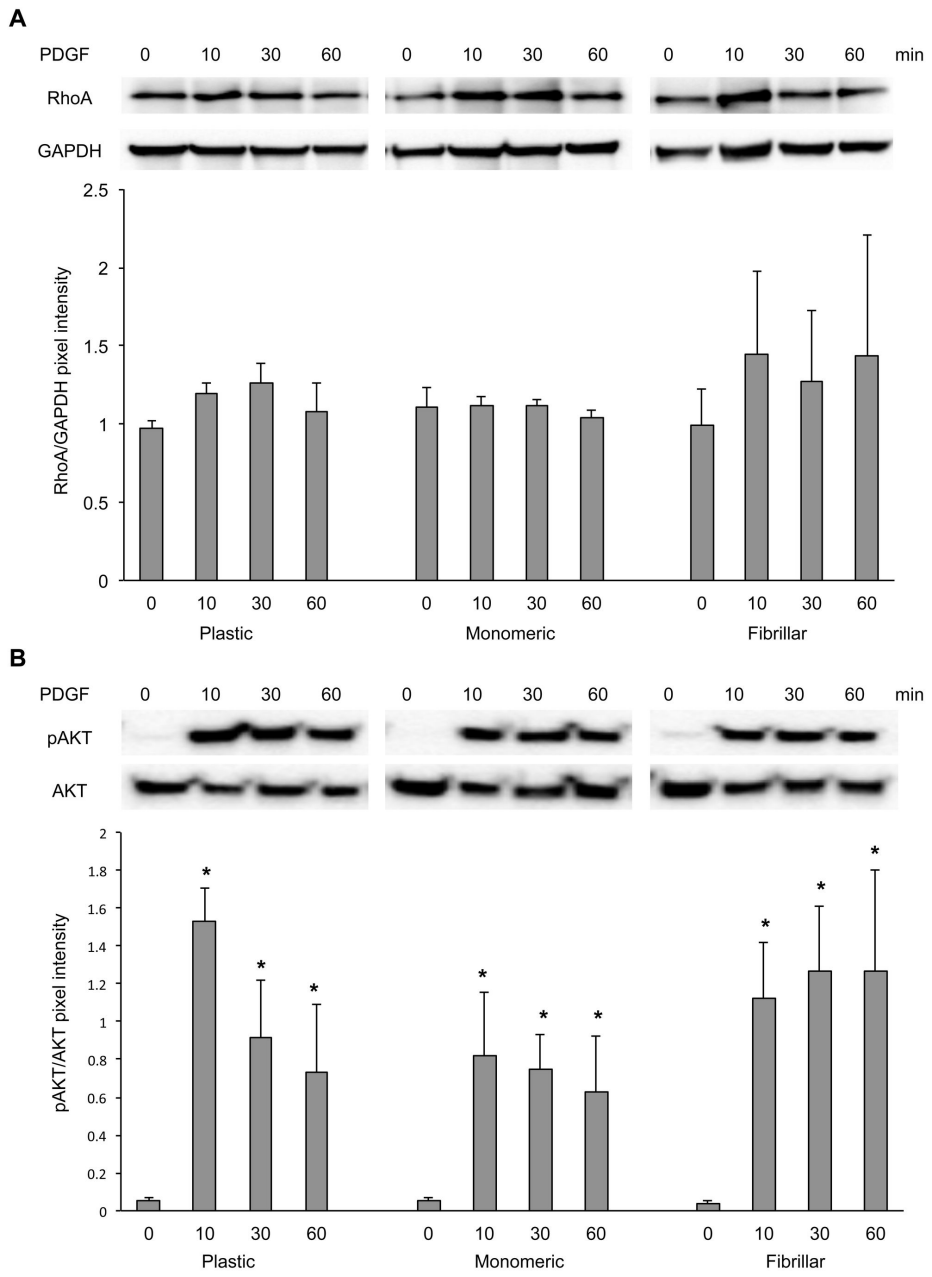
Interaction of LSMCs with ECM is not modulated by RhoA or AKT. LSMCs cultured on plastic, monomeric or fibrillar collagen-coated dishes were treated with 10 ng/ml PDGF for various time points and blotted for RhoA and GAPDH (A) as well as pAKT and AKT (B). Statistical significance within each matrix and compared to non-treated sample is indicated by asterisks (n =3, p<0.05).

**Figure 8:**
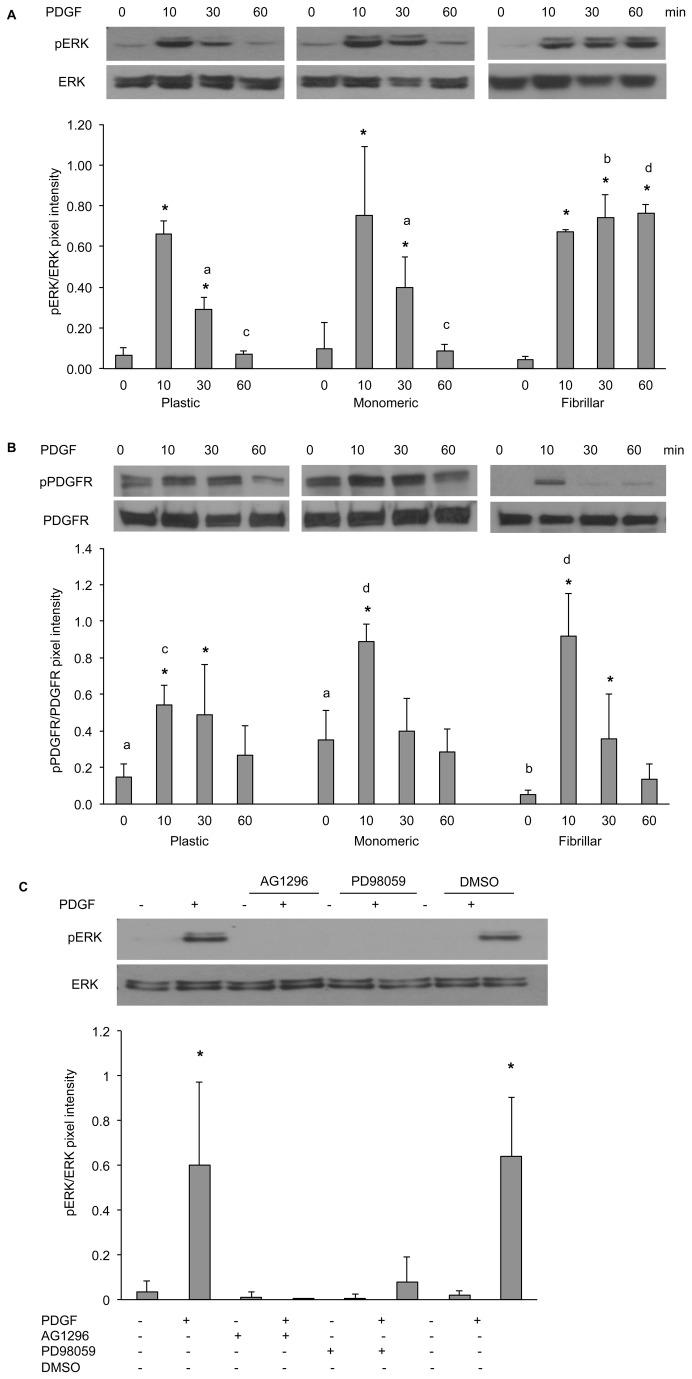
Activation of MAPK signaling pathway is influenced by the interaction of LSMCs with ECM. (A & B) LSMCs were cultured on plastic, monomeric or fibrillar collagen-coated dishes and then treated with 10 ng/ml PDGF for various time points. Lysates were blotted for (A) pERK1/2 and ERK1/2, and (B) pPDGFR and PDGFR. Densitometry values were normalized to total protein expression for each phosphorylated protein. (C) LSMCs cultured on plastic dishes were pre-treated with 10 µM of AG1296 or 25 µM of PD98059 in serum-free medium for two hours followed by treatment with 10 ng/ml PDGF for 10 minutes. Lysates were blotted for pERK1/2 and ERK1/2 Statistical significance within each matrix and compared to non-treated sample is indicated by asterisks. Letters indicate statistical differences across all samples on different matrices (n =3, p<0.05).

To elucidate the signaling mechanisms involved in the interaction of collagen matrices with LSMCs, we investigated MAPK, AKT and Rho-GTPase signaling which have been shown to be associated with the effects of ECM in a variety of cell types. LSMCs were cultured on plastic, monomeric or fibrillar collagen-coated dishes and stimulated with 10 ng/ml PDGF for 0, 10, 30, and 60 minutes. Results showed that while there was no significant difference in the activation of RhoA across matrices (Figure 7A), both AKT (Figure 7B) and ERK1/2 (Figure 8A) were strongly activated as early as 10 minutes after PDGF stimulation in LSMCs cultured on all matrices. AKT activation on all matrices persisted over time, whereas activation of ERK1/2 on plastic and monomeric collagen matrices returned to basal levels within an hour but persisted in LSMCs cultured on fibrillar collagen matrix. We next looked at the activation of PDGFR on matrices. The basal level of PDGFR phosphorylation (time 0) was lower in LSMCs cultured on the fibrillar collagen. However, a robust activation of PDGFR was observed on all matrices within 10 minutes in response to PDGF treatment (Figure 8B). This activation was significantly stronger in cells cultured on monomeric collagen than on plastic matrix, but not fibrillar collagen. Furthermore, the duration of this phosphorylation was longer on monomeric collagen than for LSMCs cultured on the other matrices.

We next determined the specificity of PDGF activation of the MAPK signaling pathway in LSMCs. LSMCs cultured on plastic were pre-treated with inhibitors specific to PDGFR and ERK1/2, AG1296 and PD98059 respectively, for two hours followed by a 10 minute stimulation with 10 ng/ml PDGF. Results showed that both inhibitors could significantly reduce the phosphorylation of ERK1/2(Figure 8C), suggesting the specificity of the MAPK signaling activation on matrices.

## Discussion

In the present study, we investigated interactions of primary human uterine LSMCs with different collagen matrices representing normal and fibrotic uterine SMCs in the absence or presence of the mitogenic growth factor PDGF. In our model, we used non-polymerized monomeric collagen matrix to mimic the ECM environment in ULs where SMCs continuously synthesize nascent collagen and collagen is also subjected to MMP-mediated degradation leading to a higher proportion of non-cross linked collagen that is laid down in a disorganized manner [7,17]. Polymerized collagen gels were used to mimic the highly ordered ECM in normal myometrial tissue. Our results showed that LSMCs cultured on monomeric non-crosslinked collagen showed increased proliferation, increased progression through the cell cycle, increased phosphorylation of FAK, decreased expression of the p21 cell cycle inhibitor, and altered cytoskeletal organization and activation of the MAPK signaling pathway when compared to cells grown on fibrillar collagen gels. These findings are similar to studies in other cell types where distinct morphologies and biological functions were observed for cells grown on monomeric versus fibrillar forms of collagens [20,21,40–42].

Cell shape and extracellular environment have been recognized as major determinants of cell behavior and function [43–48]. Cells can sense the degree of extension or compression in their surroundings to modulate specific cell processes that affect cell survival, differentiation, or apoptosis [43,49–54]. Mammary epithelial cells lose their epithelial morphology once matrix rigidity decreases below a specific threshold [55,56]. Similarly, vascular SMCs obtain a more proliferative phenotype when cultured on rigid collagen films [57]. We did not measure the exact mechanical force exerted on LSMCs from monomeric and fibrillar collagen matrices. However, the differential growth of LSMCs on the gelatinous, pliable form of the fibrillar collagen compared to the more rigid monomeric collagen coating suggests that LSMCs sense different mechanical cues on different collagen matrices. Both of these factors - matrix rigidity and pliability- are known to change the degree of mechanical stress exerted on cells and hence alter cell phenotype and function [35]. It is, therefore, possible that uterine LSMCs have a higher rate of proliferation because they are being exposed to greater amounts of monomeric or degraded collagen whereas in normal myometrial tissue, proper synthesis, assembly and turnover of collagen molecules provide cells with timely cues for quiescence or proliferation.

Mechanical stress exerted upon cells through the ECM in both normal and diseased tissues is transmitted through molecular players such as integrin receptors, focal adhesions, cytoskeletal proteins, and signaling pathways including MAPK, PI3K/AKT, and Rho-GTPase [58–60]. We observed increased phosphorylation of FAK which is regulated by the physical properties of the matrix [61], supporting the active formation of focal adhesions in LSMCs cultured on monomeric collagen matrix. It is possible that such activation, along with the pronounced expression and clustered placement of vinculin and F-actin stress fibers observed on the monomeric collagen, could lead to hyper-activation of the MAPK signaling pathway which in turn can cause an increase in the rate of cell proliferation on this matrix. In addition, involvement of Rho-GTPases other than RhoA, as well as integrins other than α2β1 integrin, should be considered as other contributing factors to the observed differences between monomeric and fibrillar collagen matrices.

The decreased level of activation or clustered localization of vinculin, FAK, and F-actin stress fibers in LSMCs cultured on fibrillar collagen observed in this study could explain the state of quiescence or decreased cell proliferation in LSMCs grown on this matrix. In fact, potent suppression of focal adhesion formation, as indicated by altered vinculin localization, has been linked to changes in gene expression and cell function in vascular SMCs cultured on polymerized fibrillar collagen [62]. Cell cycle arrest at the G0/G1-S and G2-M transition in cells cultured on fibrillar collagen has also been linked to alterations in cell proliferation [20,22,37]. In our study, we found that LSMCs cultured on monomeric collagen had enhanced progression from the G1-S phase of the cell cycle and down-regulation of the cyclin inhibitor p21 but not p27. Indeed, vascular SMCs have also been shown to have lower expression of p21 when cultured on monomeric collagen [22]. A similar phenomenon has also been reported in lung mesenchymal cells cultured on monomeric collagen as these had higher levels of cyclin E1 and CDK2 mRNA along with increased cell proliferation when compared to cells cultured on fibrillar collagen [42]. Other studies have reported that LSMCs escape G1/G0 arrest through up-regulation of cyclins D & G1 [63,64]. In addition, transfection of LSMCs with cyclin G1 antisense oligos led to increased expression of both p21 and p27 cyclin-dependent kinase inhibitors and induction of apoptosis [64]. Leiomyoma cells also showed increased proliferation in response to fenvalerate due to down-regulation of the cyclin inhibitor p27 compared to normal uterine myometrial cells [65].

In ULs an increased presence of and response to growth factors such as PDGF have been documented [66–68] along with alterations in EMC collagens. The results of our proliferation studies support such findings but the observed synergistic effect of monomeric collagen in augmenting the stimulatory effects of PDGF in LSMC proliferation, which was not present in cells cultured on fibrillar collagen, is novel. Indeed, similar observations in other cell types including vascular SMCs suggest the presence of a cross-talk between collagens and PDGF in regulating cell proliferation [31,69–76]. Moreover, stimulation of LSMCs with PDGF resulted in the activation of the MAPK signaling pathway on both collagen matrices but in distinct ways. The dynamics of PDGFR activation were similar on both monomeric and fibrillar collagen matrices in response to PDGF treatment. PDGFR activation upon PDGF stimulation was rapid and transient in LSMCs on both matrices. However, the presence of PDGFR activation in the absence of any PDGF stimulation for LSMCs grown on monomeric collagen suggests interplay between ECM/collagen receptors and PDGF/PDGFR leading to differential modulation of downstream molecules such as FAK as discussed above.

Activation of PI3K/AKT or JAK/STAT in cells grown on different matrices has been reported. In hepatocytes matrix stiffness enhanced ERK1/2, protein kinase B (PKB/AKT) and signal transducer and activator of transcription-3 (STAT3) phosphorylation and resulted in enhanced mitogenic signaling in response to hepatocyte growth factor [77]. Similar activation of MAPK and AKT pathways was observed in mesenchymal stem cells cultured on different matrices [58]. While we did not observe the involvement of the AKT pathway or αβ1 integrin in the interaction of LSMCs with collagen matrices, there remains the question of whether other signaling pathways and collagen receptors may play a role in the differential response of LSMCs on specific collagen matrices.

We also observed different activation patterns for ERK1/2 on monomeric collagen versus fibrillar collagen matrix. Rapid activation of ERK1/2 on monomeric collagen nicely followed the activation of PDGFR in response to PDGF treatment. In contrast, we observed persistent ERK1/2 activation in LSMCs cultured on fibrillar collagen in response to PDGF stimulation. The duration of ERK1/2 activity in response to growth factors has been linked to differential regulation of cellular responses and cell fate in a cell-type dependent manner. Treatment of PC12 pheochromocytoma cells with nerve growth factor caused sustained activation of ERK1/2 for 2-3 hours leading to cell differentiation whereas epidermal growth factor induced a transient ERK1/2 activation required for cell proliferation [78,79]. On the other hand, mitogenic stimulation in fibroblasts contributes to sustained ERK1/2 activation which in turn maintains decreased expression levels of anti-proliferative genes [80,81]. Mechanisms involved in transient or sustained activation of ERK1/2 are known to be modulated by both spatio-temporal regulation as well as diverse chemical and mechanical stimuli [82,83]. Activation of ERK1/2, in general, leads to progression through the cell cycle. However, there are several reports indicating that long term activation of ERK1/2 promotes cell differentiation or even apoptosis [84–86]. Such effects are mediated by upregulation of p21 and coordinated through PI3K/AKT [82,87–89]. In addition, differential sensitivity to the duration of ERK1/2 activity, stimulus and phosphatase-specific mechanisms involved in MAPK regulation, along with the involvement of the non-catalytic site in ERK1/2 may play a role in the pattern of ERK1/2 activation [90–92]. The involvement of any or all of the above-mentioned factors in LSMCs stimulated with PDGF is of importance considering that these cells were also exposed to different ECM environments and mechanical cues. A similar convergence of chemical and physical signaling by PDGF and different forms of collagen was recently reported for vascular SMCs [41]. Future investigations to identify the underlying molecular mechanisms using MAPK specific inhibitors or mutants in LSMCs are warranted.

In conclusion, our significant findings on the characterization of LSMCs cultured on different forms of collagen as a simple model to better replicate the ECM environment in normal myometrium and fibrotic leiomyoma tissue were two fold. First, the findings demonstrate the importance of carefully defining the culture systems for studying specific cell functions. This study along with others, using various cell lines including LSMCs [93,94], have shown that growth of cells on conventional plastic dishes induces cell growth and morphology that differ from the cellular characteristics induced by growing cells on ECM that is representative of the ECM surrounding cells in normal vs diseased tissue. The second more important aspect of our findings highlights the critical role of ECM collagen as a mechanical/physical stimulus for cell proliferation or differentiation and its interaction with a biological stimulus such as PDGF in the pathogenesis of ULs. Whether excess synthesis and deposition of malformed ECM collagen is the cause or subsequent consequence of UL formation, the fact that it can further modulate tumor growth, opens up new avenues for research on the use of anti-fibrotic drugs such as halofuginone and interferons for therapeutic use. Our findings provide a framework for identifying targets in the initiation and development of tissue fibrosis in myometrium for screening and therapeutic purposes.
